# *Closing the Gap*: Mechanisms of Epithelial Fusion During Optic Fissure Closure

**DOI:** 10.3389/fcell.2020.620774

**Published:** 2021-01-11

**Authors:** Brian Ho Ching Chan, Mariya Moosajee, Joe Rainger

**Affiliations:** ^1^The Division of Functional Genetics and Development, The Royal Dick School of Veterinary Sciences, The Roslin Institute, The University of Edinburgh, Scotland, United Kingdom; ^2^University College London Institute of Ophthalmology, London, United Kingdom; ^3^The Francis Crick Institute, London, United Kingdom; ^4^Moorfields Eye Hospital NHS Foundation Trust, London, United Kingdom; ^5^Great Ormond Street Hospital for Children NHS Foundation Trust, London, United Kingdom

**Keywords:** optic fissure closure, basement membrane, apoptosis, eye development, transcriptome (RNA-seq), coloboma, EMT—epithelial to mesenchymal transition, cell polarity and adhesion

## Abstract

A key embryonic process that occurs early in ocular development is optic fissure closure (OFC). This fusion process closes the ventral optic fissure and completes the circumferential continuity of the 3-dimensional eye. It is defined by the coming together and fusion of opposing neuroepithelia along the entire proximal-distal axis of the ventral optic cup, involving future neural retina, retinal pigment epithelium (RPE), optic nerve, ciliary body, and iris. Once these have occurred, cells within the fused seam differentiate into components of the functioning visual system. Correct development and progression of OFC, and the continued integrity of the fused margin along this axis, are important for the overall structure of the eye. Failure of OFC results in ocular coloboma—a significant cause of childhood visual impairment that can be associated with several complex ocular phenotypes including microphthalmia and anterior segment dysgenesis. Despite a large number of genes identified, the exact pathways that definitively mediate fusion have not yet been found, reflecting both the biological complexity and genetic heterogeneity of the process. This review will highlight how recent developmental studies have become focused specifically on the epithelial fusion aspects of OFC, applying a range of model organisms (spanning fish, avian, and mammalian species) and utilizing emerging high-resolution live-imaging technologies, transgenic fluorescent models, and unbiased transcriptomic analyses of segmentally-dissected fissure tissue. Key aspects of the fusion process are discussed, including basement membrane dynamics, unique cell behaviors, and the identities and fates of the cells that mediate fusion. These will be set in the context of what is now known, and how these point the way to new avenues of research.

## Introduction

Tissue fusion is an essential process in vertebrate development. It requires the growth and coming together of groups or sheets of cells, typically epithelia, and their joining to create continuous bodies with lasting structural integrity (Ray and Niswander, 2012). Fusion occurs throughout embryogenesis, in well-studied contexts such as the heart, neural tube, and palate, but it also occurs in less well known settings such as the genitalia, esophagus, avian lungs, face, eyelids, and body wall (Van Der Werff et al., 2000; Williamson et al., 2006; Sadler, 2010; Ray and Niswander, 2012; Palmer and Nelson, 2017, 2020). Not all fusions are the same, and each has subtle nuances that confound broad advances in our understanding of tissue fusion mechanisms, such as the range of cell types involved, the orientation of cells at the fusing edge, local mechanical forces or stresses on growing tissues, and the presence or absence of encapsulating basement membranes. Thus, fusion is a developmental and cell-behavioral enigma. Here, we focus on the process of fusion during OFC, the developmental closing of a gap in the early embryonic eye. Fusion mechanisms during OFC are of particular importance due to the clinical relevance of OFC defects and poor understanding of their causality, and as with all fusion defects—prevention through better understanding is the ultimate objective.

## What Is Optic Fissure Closure?

**FIGURE 1:**
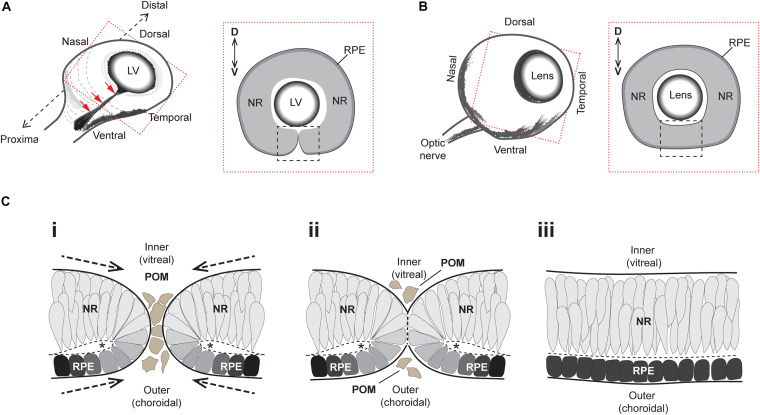
Superficial processes of optic fissure closure. **(A)** *Left*: Cartoon of the early 3D optic cup with a ventral cleft—*the optic fissure*—running along the entire proximal-distal axis (red arrows). *Right*: Slice-section cut perpendicular to the P-D axis and at the midpoint (hatching in *Left*) of the optic cup depicting the optic fissure margins (black box) which at this stage are apposed but not fused. **(B)** *Left*: Later staged optic cup with a now fully fused optic fissure. *Right*: Slice-section shows the fused optic fissure (black box) has completed the circumferential continuity of both neural retina and the RPE in the ventral optic cup. **(C)** Sequence of key cellular and morphological events during optic fissure closure: **(i)** The nasal and temporal OFMs move toward each other and become spatially apposed, with only a few POM cells separating the edges of each. At this stage, the OFMs are each fully encapsulated by single basement membranes (solid black lines) and the sub-retinal space is folded over as folding points (FP, asterisks); **(ii)** contact occurs between the two OFMs and POM cells are excluded as the apposed OFMs establish a fusion plate and the separating basement membranes are displaced (thick hatched black lines). **(iii)** Fusion has occurred and the RPE and NR are each organized into epithelial continuum, while two distinct basement membranes are now established at the inner and outer regions of the optic cup. The FP has become the sub-retinal space (Hatched line). Key- LV, lens vesicle; D-V, dorsal-ventral; NR, neural retina; RPE, retinal pigmented epithelium; POM, periocular mesenchyme.

The developmental formation of the eye is broadly conserved among vertebrates and requires a complex sequence of coordinated morphogenetic events (reviewed in Chow and Lang, 2001; Fuhrmann, 2010). Early embryonic neuroepithelial tissue bilaterally evaginates from the anterior neural plate as pouch-like optic vesicles, which then meet the overlying surface ectoderm to trigger a mutual invagination of both tissue layers to form a lens and optic cup, respectively. As the optic cup continues to grow it folds around itself laterally to produce a double-layered retina structure which contains an inner neural retina (NR) layer and an outer retinal pigment epithelium (RPE) layer (Figure 1A). This structural growth results in a transient ventral cleft running along the entire proximal-distal (PD) axis of the optic cup, from the site of the future iris all the way to the head of the presumptive optic nerve (Figure 1A). This cleft is interchangeably referred to as the “choroid” or “optic” fissure (OF), and its closure (optic fissure closure; OFC) completes the circumferential continuity of the vertebrate eye, providing a structural framework for subsequent eye development (Figure 1B).

The superficial OFC process involves the meeting and fusion of opposing multi-layered epithelia from the nasal and temporal edges of the optic fissure margin (Figure 1C). These edges are initially surrounded by their own separate basement membranes and comprise a mix of NR and RPE cells attached at their apical edges to a spindle—the OF folding point—(Figure 1Ci). The OF margins come closer together as the eye cup grows, eventually becoming completely apposed with basement membranes (BM) from both edges coming into direct contact with each other (Figure 1Cii). The lips of the fissure meet broadly at the center of the optic cup and the fusion process extends proximally and distally until the entire OF is fused. The BM is breeched or partially removed to permit the mixing of cells from either edge. Subsequently these cells reorganize and integrate with the distinct and continuous epithelial layers of NR and RPE, with the formation of two BM layers at the inner (vitreal) and outer (choroidal) aspect of the ventral eye (Figure 1Ciii). Once fusion has occurred, the ventral retina is indistinguishable from the adjacent retina, and differentiation of the multiple retinal cell types and maturity of RPE cells can subsequently occur.

## Coloboma Is a Failure of OFC

**FIGURE 2:**
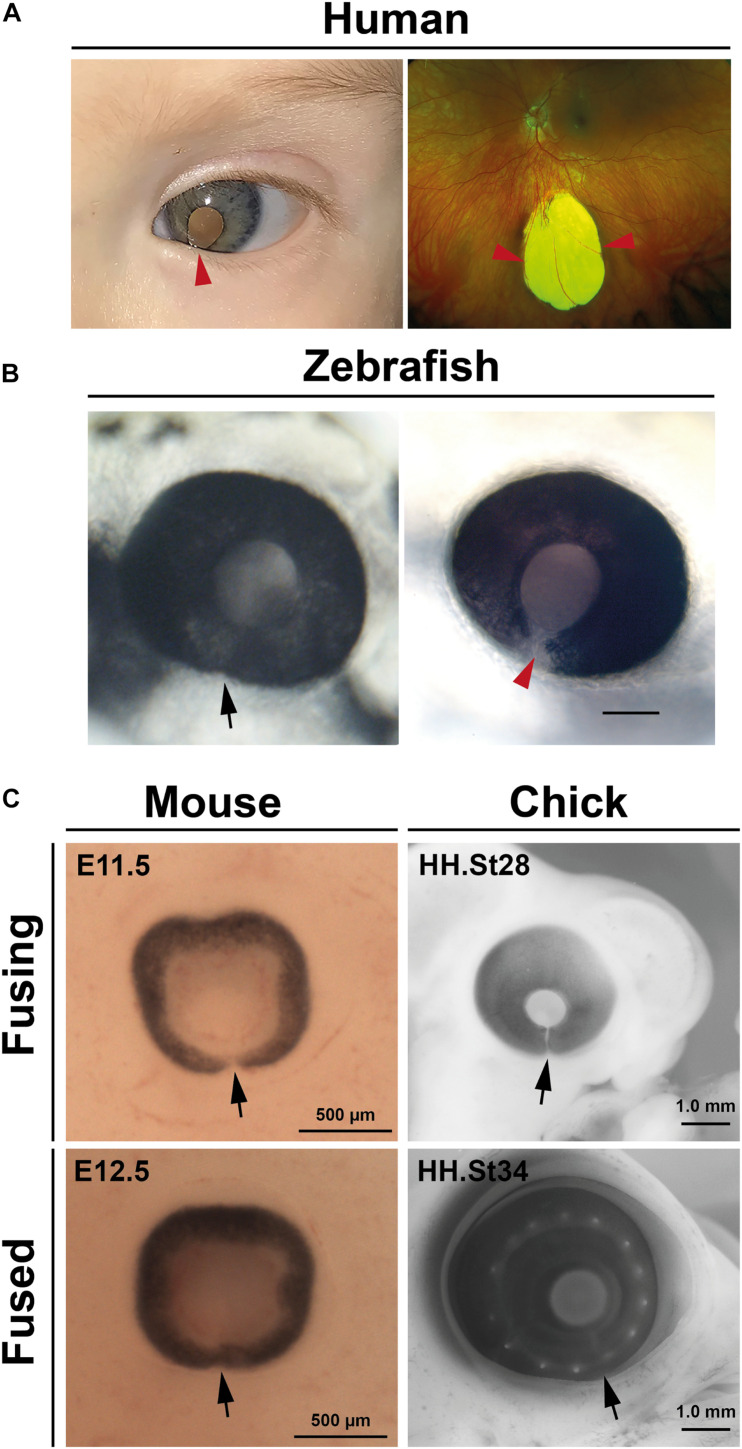
Coloboma and optic fissure closure. **(A)** Human colobomas. Anterior segment photo of the left eye showing iris coloboma and white reflex through the pupil (left) and Ultrawidefield color fundus imaging of the left retina showing an inferior chorioretinal coloboma not involving the optic disc or macula (right). Arrowheads indicate affected regions. **(B)** Fused optic fissure in zebrafish (left; arrow) and persistent colobomatous defect at 56 hpf (right; arrowhead). **(C)** Optic fissures (arrows) in the eyes of mouse and chick embryos at fusing and fused stages.

Failure of OFC is clinically referred to as ocular (or uveal) coloboma (OC) and manifests as open clefts at any point along the ventral PD axis of the eye (Figure 2A). OCs can vary in appearance and severity of the ocular tissues affected: at the distal-most region of the eye the unfused inferior iris may lead to a keyhole pupil shaped gap which may have only mild effects with photophobia; whereas OC involving the retina, choroid, RPE, or optic nerve can impair vision (the superotemporal field which corresponds to the inferonasal location of the defect), including central vision if the macula is also involved (reviewed in Gregory-Evans et al., 2004; Chang et al., 2006; ALSomiry et al., 2019). OC sits within a phenotypic continuum of developmental eye defects with microphthalmia (small eye) and anophthalmia (little-to-no ocular tissue), which are collectively referred to as the “MAC” spectrum of structural eye defects. MAC can affect either one or both eyes, can vary asymmetrically in severity, and can occur either in isolation, with other ocular features such as anterior segment dysgenesis (ASD) or cataract (complex) or as part of a wider developmental syndrome (Williamson and FitzPatrick, 2014; Patel and Sowden, 2017). Although non-genetic causes of OC have some basis in epidemiological evidence, the disorder is considered to be largely genetic (Morrison et al., 2002; Gregory-Evans et al., 2004), with over 40 genes so far identified harboring causative mutations in OC patients (for thorough reviews of the genes implicated in human colobomas—see Patel and Sowden, 2017; ALSomiry et al., 2019; George et al., 2020; Yoon et al., 2020).

Knowledge of coloboma-gene function in eye development is well supported by animal models using knock-out or engineered disruptions to disease genes. The vast majority of these loci encode transcription factors or signaling molecules that drive the early phases of eye development, such as patterning and growth, thereby implicating these genes for roles in setting up the eye to be competent to fuse (Chow and Lang, 2001; Patel and Sowden, 2017; ALSomiry et al., 2019). In many of these, the fissure margins do not become opposed and cannot make adequate contact for fusion. However, despite extensive DNA sequencing of coloboma patients and families the majority of isolated OC affected individuals (>80%) remain without an identified genetic cause. Genetic heterogeneity is a major confounding feature as no single genetic locus accounts for more than 3% of cases (Williamson and FitzPatrick, 2014; Rainger et al., 2017; Jackson et al., 2020). Failure of epithelial fusion is an obvious mechanism that may account for this lack of molecular diagnoses for OC, as the cellular behaviors and processes that mediate fusion and/or maintain tissue integrity at the fused fissure are as elusive as the genetic factors that regulate them. In combination these have major implications for our understanding of human eye development and are likely to have wider relevance for other developmental contexts that involve tissue fusion. Some recent publications have begun to address these issues with particular focus on the biology at the optic fissure itself. This timely review will therefore focus on what is known about the developmental mechanisms of fusion during OFC, will expose the areas where knowledge gaps remain, and will provide a useful framework to direct future research, including predicting emerging technologies that can be utilized to make transformative breakthroughs in this area.

## Animal Model Systems Defined Key OFC Events

Much of our current knowledge of OFC progression or coloboma causation has come from studies in fish, avians, and rodents, with the vast majority of these most recently carried out using zebrafish or mouse (Figures 2B,C). These have also been cross referenced to knowledge attained from studies of fixed human tissues (Mann, 1964; O’Rahilly, 1966). Superficially, processes appear to be highly conserved among all species so far analyzed and are discussed in depth in later sections: fusion initiates at a single point in the midline of the ventral optic cup and extends in both proximal and distal directions, with fusion complete in embryonic stages and prior to the onset of most retinal cell-type differentiation.

Mouse studies of specific gene functions predominated when gene knock-out technologies or mutagenesis screens were developed, but these were largely superseded through the use of morpholino gene knock-down, and latterly gene-editing approaches in zebrafish. In this context these genetically tractable species have been vitally important to determine the conserved patterning events at the ventral retina and optic stalk, and the proportional growth of the optic cup that occurs to facilitate the correct apposition of the OF margins prior to the onset of fusion (Patel and Sowden, 2017; ALSomiry et al., 2019). They have also proven vitally important in confirming the pathogenicity of human coloboma variants.

In zebrafish, the use of fluorescent reporter systems coupled to live-imaging confocal microscopy has been extremely powerful to determine specific cellular behaviors during OFC (Gestri et al., 2018; Eckert et al., 2020). Two new studies have recently established chicken embryos (Figure 2C) as models for OFC research, both with particular focus on fusion events (Bernstein et al., 2018; Hardy et al., 2019). The timings of OFC have now been established for all three species and highlight some distinct advantages of the chick OFC system, including the broadest temporal window for experimentation or analysis (Table 1). In addition, the chick OF is structurally larger and therefore provides more OF tissue for analyses than either mouse or zebrafish. However, the major experimental drawbacks of both chicken and mouse embryo models are the current lack of OFC live imaging modalities, a distinct feature of zebrafish work. Explant cultures and live imaging systems are available for palate and other explant cultures in these species and therefore should be amenable to develop for the optic fissure. Finally, although the *in ovo* development of chick embryos lends itself to experimental and transient genetic manipulations (e.g., electroporation and virally-mediated delivery), it has lagged far behind zebrafish and mouse for germline or high-throughput genetic studies. Coincident with the emergence of gene editing, major improvements have been made in transgenic biotechnology, potentially enabling rapid and cost effective generation of transgenic chick embryos (Taylor et al., 2017; Woodcock et al., 2017; Davey et al., 2018).

**TABLE 1:** Key timing for initiation and progression of OFC in multiple class of vertebrate.

Species	Fusion initiation	Fusion completion	Total fusion (approx.)
Chicken	HH St27-28/5.5-6 d*	HH 34/8 days	66 h
Zebrafish	34–36 hpf*	56 hpf	26 h
Mouse	11.5 days	13.0 days	24–36 h
Human	CS16/week 6	CS18/week 7	1 week

*Main sources: Mann (1964); Hardy et al. (2019), Eckert et al. (2020) (references: Mann, 1964; Hardy et al., 2019; Eckert et al., 2020).*

CS, Carnegie stage; dpf, days post fertilization; h, hours; HH, Hamburger Hamilton stage. (*Incubation times may vary between laboratories or aquaria).

## Which Cells Initiate Fusion?

There is little data to precisely determine the temporal initiation and progression of fusion during *human* OFC, reflecting a scarcity of samples and appropriate ethical considerations. Much of our knowledge dates from studies of 50 years ago or more, with observations concluding that human OFC occurs between the fifth and seventh weeks of development (see Table 1), initiates centrally, and continues bi-directionally along the P-D axis (Mann, 1964). BM breakdown is not well annotated in these studies, and cell mixing is the predominant observation of fusion. Histological plates from O’Rahilly (1966) indicate that the medial-proximal region fuses first at Carnegie Stages 16–17, as judged from proximity to the lens vesicle and its structure within the same images. Whereas fusion in the distal most optic cup and optic nerve has not yet occurred by the same stage.

In mouse, OFC is reported to start in the proximal-central portion of the OF at embryonic day E11.5–E11.75 and continues in both directions along the P-D axis, where both the iridial and optic disc regions of the OFM are the last to fuse by late E12.5–13.0 (Hero, 1989, 1990; Figure 2C).

Closure in *zebrafish*, as defined by BM breakdown, can be observed from as early as ∼34 h post fertilization (hpf) at the medial-proximal region of the optic cup, then extends bidirectionally along the P-D axis (James et al., 2016; Gestri et al., 2018). Cell mixing and fusion of the epithelial layers appears to lag several hours behind BM removal in zebrafish (James et al., 2016). Recently, fusion was shown to occur closer to the optic nerve head in the proximal aspect of this axis (Eckert et al., 2020). The length of the zebrafish fissure is approximately 60 μm and its closure is largely completed by 48 hpf, with the distal-most region being the last region to fuse by 56 hpf (Figure 2B). However, it should be noted that precise timings may vary between aquariums.

In the *chicken* embryo, a recent analysis concluded that closure initiated with a single zone of epithelial fusion in the mid-point of the optic fissure at around embryonic day (E) 6 (HH stage 28), and that closure involved simultaneous BM dissolution and cell mixing (Hardy et al., 2019). Chick OFC then continued from this region in both proximal and distal directions to include approximately 1.6 mm of fused epithelia over 60 h of fusion (Hardy et al., 2019; Figure 2C). A previous report had shown that OFC occurred earlier (HH st24, approximately E5) in the proximal region of the chick eye (Bernstein et al., 2018), but that this fusion included intercalation of astrocytes and optic nerve cells and did not show any complete joining of epithelial layers (RPE and NR), and also occurred in a region where the *pecten occuli*—a large homeostatic structure that juts into the vitreous—is embedded. Whereas fish and mammals display complete fusion along their P-D axis, a distal region of the chick fissure remains open in the iris to allow hyaloid-like blood vessels to enter and exit the eye throughout the lifespan of the eye (Hardy et al., 2019). Nevertheless, the medial region of the chick OF displays highly similar processes to human and mouse OFC involving complete bi-directional fusion of the two epithelial layers (NR and RPE). In summary, in of all these species the initial point of fusion occurs in the central region of the optic fissure and progresses bi-directionally along the PD axis.

**FIGURE 3:**
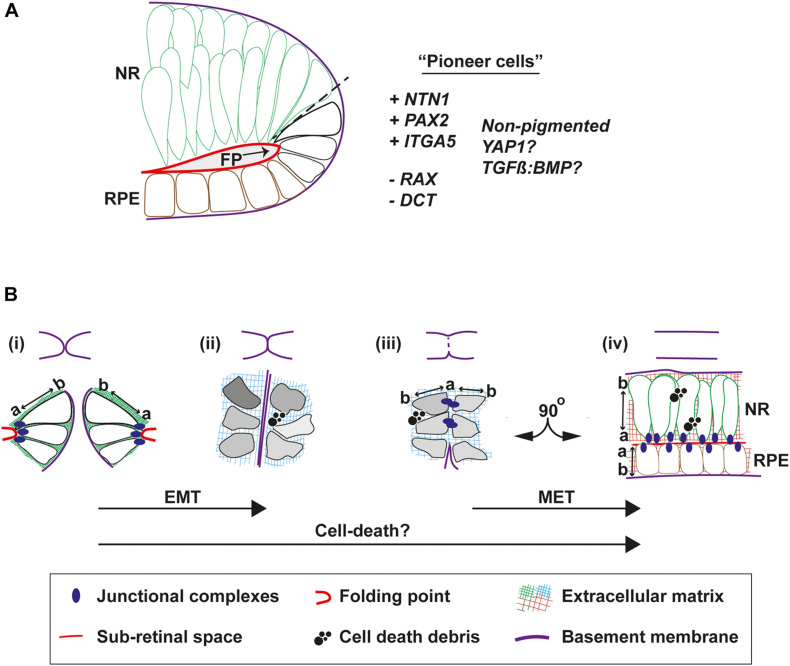
Cell behaviors that mediate fusion in the OF. **(A)** Pioneer cells are located at the edges of the fissure margins in a position intermediate between the RPE and neural retina cells. They are negative for the neural retina marker Rx:GFP and for the RPE marker DCT, but are predicted to be positive for NTN1, PAX2, and Integrin-5A. They also remain unpigmented throughout fusion. **(B)** Morphologies and behaviors of pioneer cells throughout fusion. *Top*, cartoons to depict progression of fusion. *Bottom*, **(i)** Just prior to fusion, pioneer cells at the edge of the fissure margins are tightly anchored to the folding point through junctional complexes at their apical sides, with clear apical-basal (a-b) polarity. **(ii)** As the margins are tightly apposed their BM come into close contact. Cells lose their a-b polarity and become mesenchymal in appearance, possibly triggered by compositional changes to the ECM or physical forces. **(iii)** As the BM is removed and cells on either side make contact, transient junctional complexes are present between the apical aspects of pioneer cells. **(iv)** As fusion is completed, cells reorientate through 90^o^ and align their junctions to the sub-retinal space. Debris from cell death is a feature frequently observed during normal OFC in chick and mouse, but not in zebrafish fissures. Key: EMT, epithelial to mesenchymal transition; MET, mesenchymal to epithelial transition; NR, neural retina; RPE, retinal pigmented epithelia; a, apical; b, basal; FP, folding point.

The architecture and molecular status of the epithelia in the area of fusion initiation is important in the context of accurately determining the underpinning biological mechanisms that mediate the process. In all species, neural retina cells occupy the inner (vitreal) aspect of the edges of the pre-fusion fissure margin, whereas the RPE is positioned at the outer aspect (choroidal) (Figure 1Ci). In human eyes the point of fusion, the “fusion plate,” is first seen at a position broadly at the midpoint or junction of NR and RPE cells (O’Rahilly, 1966). The fusion plate involves similarly positioned cells in chick OFC, including NR and inverted RPE cells that remain non-pigmented until well after fusion is complete (Hardy et al., 2019). During zebrafish OFC, a blood vessel (the hyaloid) is positioned at the analogous region and consequently the first contact between the margins is slightly higher (more dorsal) in this species, in the upper third of the fissure (Gestri et al., 2018; Eckert et al., 2020). These cells had flattened morphology just prior to fusion, consistent with RPE cell morphology, although they were not pigmented. These “pioneer cells” as the authors named them, became more cuboid before contact (Eckert et al., 2020) then displayed amorphous elongated shapes with protrusions and overt polarization toward the edges of the fissure margin (Gestri et al., 2018). Molecular analyses are still ongoing to precisely determine the characteristics and identity of these cells (Figure 3A), however, cell labeling and live imaging revealed they were negative for the NR progenitor reporter *Rx2:GFP* before and during fusion, but then gained *Rx2:GFP* expression in the fused margin (Eckert et al., 2020). This suggests that although these cells may appear to be of RPE origin they can acquire a NR identity. However, it also indicates that pioneer cells could be a separate novel cell type within the OF. Indeed, the RPE marker dopachrome tautomerase (*dct*) was not observed in these cells (Eckert et al., 2020). Although *Netrin1a* and *integrina5* have been observed in this location in zebrafish (Lupo et al., 2011) and this is a known region of localized *PAX2* expression, it will be important to find specific markers for pioneer cells that can be used to perform robust characterization of their contributions to fusion (are they essential?) and their fate in the fused margins, and whether these are conserved across all species. It will also be important to reveal their origin, and how and when during eye development they are specified. Their identification has the potential to be transformative in OFC research, as they represent the emergence of a tractable system that can be manipulated to resolve fusion-specific mechanisms within the complex environment of the developing eye at unprecedented resolution.

## Cells at the of Margins Exhibit a Range of Behaviors to Facilitate Fusion

Accompanying fusion are some key changes to epithelial organization at the OF (Figures 3Bi–iv). Before fusion the apical surfaces of the cells lining the fissure are connected to the folding point (Figure 3B) and their basal surfaces are aligned to the BM. Live imaging in zebrafish has shown that cells from opposite OFs approach each other in a basal to basal orientation but then depolarize during fusion (Eckert et al., 2020). Using the apical junction complexes markers (ZO-1 and alpha-catenin; adherens junctions), pioneer cells were observed to detach from the folding point and became disorganized. They then transiently aligned at their apical edges at the fusion plate, before subsequently realigning their apical edges another 90^o^ along the sub-retinal space in fused tissue (Gestri et al., 2018; Figure 3B). This process can be affected by disruptions to junctional complexes, as deletion of α-*catenin* from the developing mouse eye results in accumulation failure of adherens-junction factors N-cadherin, β-catenin, and filamentous actin on the apical side of cells at the margins and the folding point, causing fusion failure and coloboma. Mutations in cytoplasmic actins (*ACTG1* and *ACTB*) and actin-remodeling factors (*TWF1* and *LCP1*) have also been identified in patients with isolated colobomas (Rivière et al., 2012; Rainger et al., 2017), although it is not clear if these manifest from defects to cell-cell adhesion or to broader effects on cytoplasmic dynamics.

How these adhesion, polarity and cell-shape changes are regulated is unknown and presents a major challenge to elucidate, based on the transience of the process. Localized physical and mechanical forces may also influence cell responses, and the Hippo-signaling factor YAP1 has been shown to sense and transduce mechanical cues to regulate transcriptional responses or cell fate decisions (Halder et al., 2012). *YAP1* is expressed in the developing mouse RPE (Williamson et al., 2014) and is required in zebrafish for RPE identity (Miesfeld et al., 2015). Mutations in *YAP1* cause syndromal and isolated coloboma in humans and fish (Williamson et al., 2014; Miesfeld et al., 2015). Therefore, it is plausible that YAP1 mechanosensory signaling may be a crucial link between the dynamic properties of cell-adhesion, ECM, and the actomyosin skeleton, and the cell behaviors and responses within the OF.

The changes to cell shape and apical-to-basal polarity during OFC are reminiscent of epithelial-mesenchymal transition (EMT) (Lamouille et al., 2014), which has been better characterized in other fusion contexts such as palate closure, wound healing and fusion of the endocardial cushions in the developing heart (Ray and Niswander, 2012). In chick, pioneer cells appear to lose their epithelial characteristics and become almost mesenchymal in appearance as they decouple from the folding point and mix between OFMs at the fusion plate (Hardy et al., 2019). As such, the mixing of mesenchymal-like cells during fusion could be considered an invasive phenotype. If EMT is a feature of fusion, then it is likely that the opposite mesenchymal to epithelial transition (MET) also occurs in the subsequent alignment and re-epithelialization of the fused margin. It must be also considered that any EMT may be only partial and highly transient as no definitive mesenchyme markers have yet been shown as upregulated or specifically localized in the OF during fusion, therefore for now we propose referring to this as an EMT-*like* phenotype. Although the core set of EMT genes are not enriched in any of the existing OF transcriptomes (see later section), this may reflect limits of sensitivity in these assays, a tissue-specific EMT gene expression profile, or the degree of EMT-MET within the OFC process. As neither processes have been formally analyzed at the molecular level in any of the OFC model systems, it will be important to determine the contribution of EMT pathways and related molecules to fusion in this context, for example the localization and balance of E- and N-cadherins, and the specific suite of integrins expressed in pioneer cells and within the fusion plate and fused retina.

Transforming growth factor beta (TGFβ) signaling has a prominent role in the regulation of EMT processes in multiple developmental and disease contexts (Lamouille et al., 2014) and mutations in TGFβ signaling components cause palate fusion defects in humans and mice (Fitzpatrick et al., 1990; Sanford et al., 1997; Dudas et al., 2006; Iwata et al., 2011; Bush and Jiang, 2012), while adding recombinant TGFβ to chick explant cultures can artificially induce fusion of the secondary palate (Gato et al., 2002). TGFβ receptors are expressed within the developing OF and TGFβ2 knockout mice exhibit colobomas, where their fissure margins meet but do not fuse (Knickmeyer et al., 2018). Chemical inhibition of the TGFβ pathway SMADs also result in OF defects in zebrafish (Knickmeyer et al., 2018). One mechanism proposed for TGFβ at is to modulate local BMP signaling at the OF margins to regulate ECM remodeling, and presumably indirectly regulate EMT—there is good evidence that crosstalk and achieving a balance between BMP-TGFβ signaling is important in the regulation of EMT (Zeisberg et al., 2003). Several ECM components were significantly downregulated in the colobomatous TGFβ2 knockout mice (Knickmeyer et al., 2018), and an equivalent gene-ontology analysis of differentially expressed genes in chick at the point of fusion also found ECM-related processes as the most significantly enriched ontology terms. These gene-expression changes of ECM factors in both chick and mouse provide evidence that the composition of the ECM at the fissure margin is dynamic and that this is a vital component of the fusion process, likely leading to EMT through enabling changes to apical-basal polarity, epithelial disassembly, and the shape of pioneer cells. A comprehensive characterization of the microenvionment at the OF during fusion will allow a more integrated understanding of how these influence fusion processes through cellular responses.

Cell-death has an important role in multiple tissue fusion events and it is an important “final” modulator of tissue morphology in embryonic development (Haanen and Vermes, 1996; Ray and Niswander, 2012). One suggestion is that cell death triggers BM breakdown, but this link has not yet been confirmed with regards to cause and effect. Another possibility is that OF cells may be inadvertently tipped toward cell death as they detach from their surrounding neighbors or as the ECM changes in composition. These cells may fail to adequately interpret the new cues they are faced with and decide to die. However, it is still not clear to what extent programmed cell death contributes to fusion during OFC and there appear to be differences among species in this regard. Dying and phagocytic cells are observed in the fissure margins in mouse embryos (Hero, 1990), but apoptotic cells are also widely distributed throughout the whole developing eye (Ozeki et al., 2000). In zebrafish, apoptotic cells were not observed in the fissure throughout the closure process (40 h to 56 hpf), either by using Terminal deoxynucleotidyl transferase dUTP nick end labeling (TUNEL) analysis staining or live imaging (James et al., 2016; Gestri et al., 2018). Furthermore, fusion proceeded normally in a macrophage-deficient zebrafish line with no observed increase in apoptotic foci (Gestri et al., 2018). In contrast, immunofluorescence for activated-caspase-3 (A-Casp-3) in chick revealed a localized enrichment for apoptosis at the fusion plate and in the adjacently fused seam, where A-Casp-3 was specifically observed in regions where pioneer cells would be positioned if reintegrating into the newly forming epithelia (Hardy et al., 2019). A formal survey of apoptosis during human OFC has not yet been conducted, although A-Casp-3 foci were observed throughout the retina and RPE in 6–7 week old embryos (Božanić et al., 2003). The lack of observable cell death in wild-type zebrafish may reflect its non-requirement for OFC, or could be due to lack of sensitivity of the assays employed and the short time-frame of fusion progression combined with the small number of cells mediating the process. However, correct regulation of cell death has been shown to be vital for OFC in zebrafish and to be a downstream effect of the important coloboma-associated transcription factors PAX2 and VAX (Viringipurampeer et al., 2012). These combine to directly regulate the fas-associated death domain (*fadd*) gene and maintain the balance between cell death and proliferation. In this context, it was the necrosis pathway rather than the apoptotic pathway that controls cell death. Deletion of *FADD*, a key regulator of apoptosis, is suggested to cause coloboma in humans (Gregory-Evans et al., 2007). The anti-apoptotic factor *Bcl6*, through interaction with the human syndromal coloboma gene *BCOR* (Ng et al., 2004), represses p53-dependent apoptosis in the zebrafish retina (Lee et al., 2013). *Bcl6* was expressed in the OF prior to fusion and its targeted knockdown caused increased p53 expression and apoptosis at the OF, and resulted in highly penetrant colobomas in both fish and Xenopus (Lee et al., 2013). Conversely, hyperactivation of p53 in mice resulted in an increased rate of apoptosis in the retina and colobomas (Van Nostrand et al., 2014). Furthermore, p53 was shown to be negatively regulated directly by CHD7 binding at its promoter. Mutations in the *CHD7* gene are the leading cause of CHARGE syndrome (George et al., 2020), providing a link between colobomas seen in CHARGE syndrome to a direct mediator of apoptosis. These studies provide useful elucidation for a gene regulatory network in which *PAX2* and *VAX* act upstream of *fadd* and *Bcl6* to regulate the balance between cell death and cell survival in the developing ventral eye. Similarly, the ephrin A5-EphB2-JNK signaling pathway has been shown to balance the induction of apoptosis and inhibition of cell proliferation during mouse OFC (Noh et al., 2016), while reduced proliferation in the ventral eye of mice lacking *Fgfr* 1 and *Fgfr2* resulted in colobomas in the absence of changes to apoptotic cell number (Chen et al., 2012). However, none of these studies presented data to show the effects on apoptosis in cells at the fissure margin, or how they caused dysregulation of fusion *per se*. Therefore, it seems that while cell death is important for maintaining the correct number of cells within the ventral retina and/or OF to permit correct OFC, it may not play a direct role in the fusion process itself.

One question that emerges from the chick data is whether pioneer cells are actively removed from the fused epithelia by apoptosis. If disrupted, this could be a mechanism for coloboma causation, e.g., via damage to epithelial integrity of the recently fused retina. Together, these findings indicate that the requirement for cell death in OFC is not universal across different organisms, and that it may not act to mediate fusion directly.

## What Becomes of the Basement Membrane During OFC?

**FIGURE 4:**
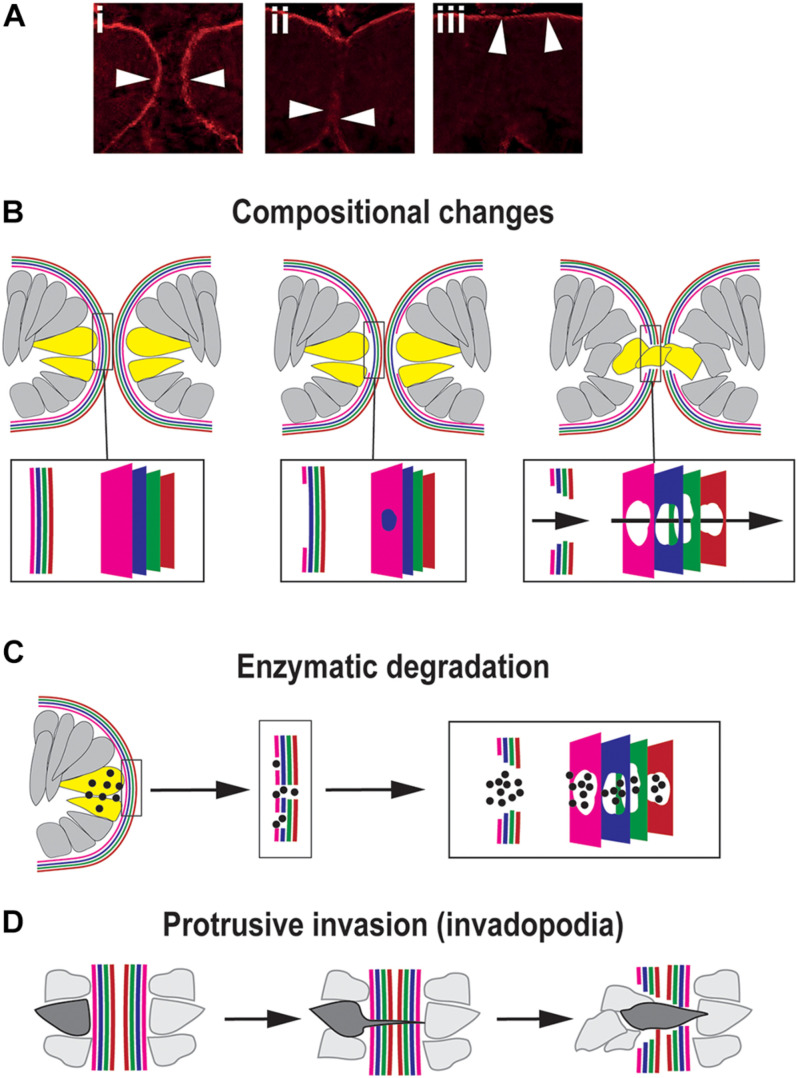
Mechanisms for traversing the basement membrane during OFC. **(A)** Dynamic changes to the localization of the BM (arrowheads) during chick OFC, as defined by antibody staining to laminin (β1). In **(i)** the OF edges are completely encapsulated by BM, in **(ii)** the BM has been locally removed or displaced at the fusion plate and cells are mixing between the two margins, and in **(iii)** the BM is now visible at the inner limits of the neural retina and the outer limit beneath the RPE. Adapted with permission from Hardy et al. (2019). **(B)** Compositional changes to the BM driven by transcriptional changes in cells at the edges of the OFM. 4x colors are used to depict the four components of BMs: collagen, laminin, HSPG and nidogens, however, the correct orientation or alignment of these in the OF-BM are not known. **(C)** Enzymatic degradation occurs through local transcription of diffusible proteases (spots) of the MMP or ADAMTS classes expressed by cells (yellow) at the edges of the fissure proximal to BM breaching. **(D)** Protrusive invasion through intact BM by actin-rich cellular protrusions.

Basement membranes (BM) are highly cross-linked and dense sheet-like structures of extracellular matrix molecules that border all epithelia (Kelley et al., 2014). The constituent molecules of the BM are predominantly large, insoluble, secreted proteins and include the core factors laminin, collagen, heparin sulfate proteoglycans (HSPGs), and nidogens (Yurchenco and Schittny, 1990; Kelley et al., 2014), although the presence and distribution of these proteins and their isoforms or paralogs may vary between different tissues. During closure, as the OFMs become apposed at their basal surfaces this positions their respective BM into direct contact with each other (Figure 4A). To enable the subsequent mixing of cells from the opposing OFMs, the BM must first be successfully breached (Figures 4Ai–iii). Several animal models of dysregulated signaling (growth factors and transcription factors) result in colobomas and exhibit a failure of BM breaching, as determined by intact BM generally shown by laminin staining (Macdonald et al., 1997; Barbieri et al., 1999; Sehgal et al., 2008; Chen et al., 2012; Cai et al., 2013). However, no studies to date have accurately identified the precise sequential mechanisms for BM modulation during OFC *in vivo* in any model organism. Major breakthroughs in this area have also been hampered by reliance on fixed-tissue samples and a lack of fluorescently-tagged or equivalently-visible basement membrane components. In combination this means the actual mechanisms for BM displacement during OFC remain largely theoretical and could be either transcriptional changes, physical removal or rupture, biochemical dissolution, or a combination of all three processes.

Transcriptional changes may be an obvious mechanism for compositional changes to the OF-BM to permit fusion (Figure 4B). At its simplest would require the time-specific reduction or change in gene expression levels of key BM components in adjacent cells, causing a subsequent reduction of synthesis for that molecular component. This would then trigger a loss of BM integrity and permit subsequent fusion events. A recent study in the zebrafish OF comprehensively analyzed all of the typical BM constituents using whole-mount *in situ* hybridization, and confirmed the expression of *collagen IV a1* and *a2; laminin a1, a4, b1a, c1*, and *c3; nidogen 1a, 1b*, and *2a;* and *perlecan* during OFC. Of these genes, only nidogen expression was observed to be down-regulated at the onset of fusion, with nidogen protein becoming absent from the BM prior to laminin removal and fusion (Carrara et al., 2019). The authors suggested that nidogen removal could be a key requirement to initiate disassembly or remodeling of the BM for fusion. This was the first identification of a BM gene with expression specifically reduced in the optic fissure margins during closure. Although loss of *laminins a1*, *b1*, and *c1* also resulted in colobomas in a separate zebrafish study (Lee and Gross, 2007), the reduction of these caused gross retinal morphogenesis defects, suggesting a broad requirement of these factors throughout eye development rather than fusion-specific roles. The observation that only *nidogen* was down-regulated out of all of the BM genes studied in the zebrafish optic fissure provides new and direct evidence to support the hypothesis that the OF-BM has a distinct and dynamic molecular make up during OFC that facilitates or permits fusion. Loss of this single factor may therefore be sufficient to trigger the onset of BM changes that are necessary for fusion. This important finding could help elucidate the subsequent events that allow for further BM modulation during OFC. RNAseq analyses of chick OFMs indicate similar transcriptional changes may occur in other species (Hardy et al., 2019). Reduced *LAMA4*; *LAMB4* and *LAMA2*; *COL4A3* and *NID2* expression were observed in the OF during closure stages when compared to the whole eye (Hardy et al., 2019). The significance of this to the structural composition of the chick OF-BM is not yet clear, and these findings require immunological or proteomic analyses to confirm the changes at the protein levels and more in depth analysis of BM composition in the region. Similar transcriptional studies in mouse and human OF tissues would be hugely beneficial to establish the conservation of such changes among vertebrates.

Expression of factors other than the core BM components may also have key roles in the fusion process. The recent identification of Netrin-1 as essential for OFC is intriguing given the localization of its protein during the fusion process and its molecular similarity to other BM components. Netrins share structural similarity to laminin N-terminal (LN) domains, contain laminin EGF-like domains, and can form heterotypic complexes with laminins and other laminin-interactors at cell surfaces, such as integrins and dystroglycans (Yurchenco and Wadsworth, 2004). The disruption of cell surface interactions with ECM and BM may be an additional mechanism for BM modulation during OFC. In mouse, chick, and human embryonic eye tissues NTN1 was observed in the BM immediately surrounding the fissure margins but did not extend far beyond this region in either direction. In addition, its transcription was significantly down-regulated immediately after fusion in all of these species (Hardy et al., 2019; Richardson et al., 2019). These suggest a fusion-specific function of NTN1 may be to somehow structurally modulate the BM. Although NTN4 has been shown to directly incorporate into laminin complexes and modulate its polymerization, NTN1 does not perform this function (Schneiders et al., 2007). In mice completely lacking *Ntn1* the fissure margins were normally decorated in Laminin and came into direct contact, but fusion did not initiate and led to highly penetrant colobomas (Hardy et al., 2019). These animals also exhibited craniofacial and inner-ear fusion defects, embryonic locations where NTN1 is similarly localized to the BMs (Salminen et al., 2000; Nishitani et al., 2017; Hardy et al., 2019). Therefore, in contrast to a down-regulation of nidogen triggering fusion, it may be that OFM-specific *NTN1* expression is required to change the molecular composition of the OF-BM to permit fusion. As a next-step, ultrastructural studies would be useful to determine if NTN1 molecules are incorporated directly within the BM or are localized to interfaces between the BM-ECM or BM-cell surface as regulators of adhesion. Similarly, thorough analysis of the compositional changes to the OF-BM in Ntn1 deficient animals could provide key information for its role in BM modulation. Another candidate BM factor is the secreted calcium-binding factor *SMOC1* (*Sparc-related modular calcium binding 1*). SMOC proteins are localized to BMs in various developmental contexts (Vannahme et al., 2002, 2003), and SMOC1 has been suggested as a fusion-specific BM-modulating factor due to causative mutations identified in patients and animal models with colobomas (Rainger et al., 2011; Patel and Sowden, 2017). *SMOC1* is specifically upregulated in the fissure region throughout closure for all species so far studied (Brown et al., 2009; Abouzeid et al., 2011; Okada et al., 2011; Rainger et al., 2011; Richardson et al., 2019). However, its role has not been determined in this context and it has yet to be found whether it has roles other than its known functions in regulating BMP signaling (Vuilleumier et al., 2010; Thomas et al., 2017; Mateus et al., 2020).

Another proposed mechanism for BM modulation is through biochemical degradation, e.g., dissolution of BM structures by extracellular proteolytic enzymes (Figure 4C). The strongest evidence for structural BM dissolution is from mouse, where ultrastructural analyses clearly showed that the BM was fragmented into multiple foci at the fusion plate (Hero, 1990). Many studies also conclude that BM is removed by this process through interpreting immunological staining for the localized absence of core BM components, typically laminin. However, few dissolution agents have been directly implicated in OFC, either through animal models with coloboma, transcriptome analyses, or human genetics. In this model, any changes to the BM must be tightly restricted regionally to ensure the integrity of the adjacent (non-OF) BM and must be also temporally restricted to ensure the BM is broken down at the right developmental moment. This would require exquisitely controlled transcription of enzymes in small numbers of cells directly at the OF margin that are likely to be below the detection thresholds of currently published OF transcriptomes, an obvious reason for why candidate enzymes have not been discovered using this approach (Brown et al., 2009; Hardy et al., 2019; Richardson et al., 2019). One factor that has recently been directly implicated is the metalloproteinase ADAMTS16 (a disintegrin and metalloproteinase with thrombospondin motifs), which was shown to be specifically expressed at the edges of both mouse and zebrafish optic fissures during closure using *in situ* hybridization studies (Cao et al., 2018). Morpholino knock-down of *adamts16* in zebrafish embryos showed coloboma causation and a failure of BM breakdown, as indicated by continual laminin and fibronectin staining at stages beyond when fusion would normally have occurred (Cao et al., 2018). However, the specificity of adamts16 mediated BM breakdown was confounded by additional changes to proliferation, cell survival and proliferation, and FGF expression at the OFM. Therefore, a precise requirement for ADAMTS16 in OF-BM breakdown is yet to be confirmed. Another class of proteolytic enzymes implicated in OFC are matrix metalloproteases (MMPs), such as those recently identified as secreted from developing hyaloid vasculature cells migrating between the zebrafish fissure margins (mmp2, 14a, 14b) and in transcriptomic studies (mmp23b) (Richardson et al., 2019; Weaver et al., 2020). Hyaloid vasculature in zebrafish is necessary for OF BM degradation, as genetic, chemical, or its physical perturbation results in a persistent BM and prevents correct OFC (Gestri et al., 2018; Weaver et al., 2020). Significantly, mmp2 was specifically implicated as functionally necessary for BM degradation in the optic fissure (Weaver et al., 2020). How these findings in zebrafish OFC translate to other vertebrates isn’t clear, and orthologs of MMPs acting in OFC in other species (or human colobomas) have not yet been identified. With the promise of transcriptomes becoming available from additional species, and the adoption of single-cell sequencing technologies in OFC models, these may reveal proteolytic enzymes and provide extra evidence for how the BM at the OF is degraded, or alternatively these may allow us to conclude that BM dissolution is not an essential OFC process that is shared among vertebrates.

A third possible mechanism for displacing the BM is through penetration and transmigration (Figure 4D). In this model a cellular protrusion forces itself through the BM at a single initial foci to enable subsequent cell migration (Kelley et al., 2014). This mechanism was first identified in cancer-line cell cultures, but in embryonic development it is employed during anchor cell invasion in *Caenorhabditis elegans*, where actin-rich cellular protrusions are referred to as “invadopodia” (Hagedorn et al., 2013). In this context, the BM is punctured and moved aside by the invadopodia of the anchor cell, rather than it being dissolved. If this mechanism is active during OFC then the protruding cell could either be from within the margin (e.g., an OFC pioneer cell) or could be extraocular (such as POM or hyaloid cell). There would then be a subsequent widening of gap in the BM as the cell pushes through and other cells mix across the layers and further displace the BM, thereby widening the gap further and mediating fusion.

Netrin receptor interactions are a key mechanism driving anchor cell invasion process (Hagedorn et al., 2013). Given the recently revealed importance of NTN1 in OFC and other developmental fusion contexts (Salminen et al., 2000; Nishitani et al., 2017; Hardy et al., 2019; Richardson et al., 2019), it is intriguing to speculate that this invadopodia function may be conserved to tissue fusion mechanisms in higher vertebrates. Although there is currently no direct evidence for this process occurring during OFC, there is some circumstantial evidence emerging to support this as a possible mechanism. Cellular protrusions have been observed at foci of BM displacement in several live-imaging zebrafish studies (Gestri et al., 2018; Eckert et al., 2020) and at the ultrastructural level in fixed mouse OF tissues (Hero, 1990). Filopodia and membrane ruffles are also key features observed during the initiation of neural tube closure, as observed using scanning electron microscopy (Rolo et al., 2016). In OFC it remains unclear whether these protrusions occur prior to or after BM displacement, and if they therefore represent invadopodium or are the establishment of subsequent cell-cell contacts. Cellular protrusions are transient and dynamic structures and notoriously difficult to observe in traditional fixed-tissue assays, but applying a combination of scanning electron microscopy and improved live-imaging techniques may be sufficient to experimentally confirm their relevance in OFC. Indeed, live imaging using a combination of differentially fluorescently-tagged BM components and cell membranes in tandem could provide definitive evidence for the presence of invadopodia or similar functional structures and the timing of their appearance, and thus functional importance, during BM displacement in OFC.

There are several other plausible or theoretical mechanisms for how the BM may be modulated to permit fusion. For example, could physical forces be important—if there is sufficient tension or energy contained in the bend of the BM, could one single breach or minor dissolution event trigger localized collapse? Biophysical analyses of the BM in this context could be applied to address this question. Other possibilities are lateral sliding of BM components (Ihara et al., 2011) that expose weak points at the OF margins. This could occur through a combination of optic cup growth and local down-regulation of adhesion receptors that provide structural links at the cellular-ECM-BM interface.

The precise mechanisms of BM modulation remain distinctly uncharacterized and it may prove that a combination of these mechanisms act in a coordinated manner to permit OFC. Nevertheless, several key breakthroughs have begun to shed some light on this process and have set a framework for this topic to be an exciting and vibrantly active research area.

## Transcriptional Studies Contribute to the Emergence of Novel Mediators of Fusion

The establishment of the animal model systems described above have enabled the use of whole-transcriptome based gene expression analyses to determine OFC relevant genes. Specific isolation of tissue at the OF with gene expression analysis is a powerful method to identify genes with roles in fusion and coloboma candidates. This was first attempted by Brown and colleagues (Brown et al., 2009) who applied laser-capture microdissection (LCM) microscopy to mouse OFs before, during, and after fusion, and then used expression microarrays to determine gene signatures associated with OFC. Their approach revealed two novel coloboma candidate loci (*NLZ1* and *NLZ2*) and provided the first available list of relative gene expression levels in the fissure margins coincident with fusion. It took another 10 years before the use of RNA sequencing was similarly applied to provide unbiased and fully quantitative gene expression during OFC, in this case with zebrafish and chick OFs (Hardy et al., 2019; Richardson et al., 2019). In the zebrafish study, tissue was collected before, during, and after fusion. At 48 hpf, when fusion is active, 79 differentially expressed genes (DEGs) were identified; of which 63 DEGs were upregulated and 16 were downregulated in OF tissue. Due to the size of the chick OF, micro-dissection was able to more accurately separate fissure margin tissues from the broader ventral optic cup during fusion and this approach identified 101 genes that were upregulated in the OF and 437 that were downregulated. Of these, 133 genes were unannotated due to limitations of current chick genome build or unmatched to human homologs according to HGNC^1^. Of the known human coloboma-associated genes (as listed in Patel and Sowden, 2017; ALSomiry et al., 2019) only *PAX2*, *ALDH1A3* (*ALDH6*), STRA6 and *SMOC1* were found to among the genes that were significantly enriched in both chick and zebrafish datasets at the OF during fusion (Hardy et al., 2019; Richardson et al., 2019). Two other known ventral eye markers with confirmed colobomas in knock-out animal models—*VAX1/2* and *BMPR1B* (Mui et al., 2005; Yan et al., 2020)—were also significantly enriched. This is strong evidence for conservation of gene networks among these divergent species (Gregory-Evans et al., 2004; ALSomiry et al., 2019). However, all these genes represent hierarchical signaling molecules or transcription factors, while their expression levels remained at similar levels after fusion had completed (Richardson et al., 2019), suggesting their role in OFC is unlikely to be fusion specific.

Ontology enrichment analysis for zebrafish OF genes at 48 hpf did not reveal any plausibly relevant biological processes relating to fusion (Richardson et al., 2019), however similar analyses with chick OF data returned significant enrichment for cell adhesion processes [“Biological adhesion” (GO:0022610) and “cell adhesion” (GO:0022610)]. Within the group of genes assigned to these processes, several candidates for roles during OFC fusion were revealed, such as *Integrin-A2*, *Cadherin-4*, *Collagen 18A1, NTN1*, and *FLRT3* (fibronectin leucine rich transmembrane protein 3), and Netrin-1. Cadherins are important for epithelial cell-cell adhesion via adherens junctions, whereas Integrin clustering and integrin-mediated cell-anchoring to fibronectin-rich basement membrane at fusion sites were recently been shown to be essential during neural tube closure (Molè et al., 2020). Collagen 18A1 is a basement membrane protein that proteolytically produces endostatin and a frizzled motif from its C-and N-terminals, respectively. The physiological significance of these is currently unknown, but Col18a isoforms have been implicated for roles in wound healing, endothelial cell motility, and may interact with laminins, nidogens and other heparan sulfate proteoglycans (Maeba et al., 2019). Mutations in COL18A1 are also implicated in neural tube closure defects (Sertié et al., 2000). The *Netrin*-1 (*NTN1*) gene was one of the highest expressed and most fissure-specific gene throughout the fusion process in both studies. *Ntn1* had also been previously identified as up-regulated in mouse OF (Brown et al., 2009), and in all three species its expression is immediately reduced or absent in the nascently-fused margin. Targeted loss of *Netrin-1* results in highly penetrant colobomas in mice and zebrafish, with mouse embryos displaying additional fusion defects in the developing inner-ear and palate (Salminen et al., 2000; Yung et al., 2015; Hardy et al., 2019). The functional mechanisms of *NTN1* in fusion are is currently unclear, but these transcriptional approaches revealed *NTN1* as a novel OCF/coloboma gene among many other plausible candidates. Subsequent studies will undoubtably reveal their significance to fusion processes during OFC.

## Summary and Future Perspectives

The recent identification of a subset of cells that guide fusion, and the adoption of transcriptional approaches that have detected specific fusion genes, and the ability to track cells as they progress their way through fusion, have combined to provide a solid framework for elucidating the remaining mechanisms underlying fusion at the optic fissure margin (see Box 1). The challenge is to now use these advances to uncover even deeper cell behaviors and the associated genetic and/or physical cues that guide them and to integrate these with human genetic and epidemiological studies to build up a comprehensive knowledge of the process and regulation of normal and abnormal fusion. There are still several key questions to be addressed. Firstly, the pioneer cells identified in zebrafish must be robustly characterized. Their equivalents should also be identified in chick, mouse and ultimately human OF tissues to confirm their broader relevance, and their molecular signatures must be well defined in each species. The more markers that are revealed in these cells, the more we can understand their unique biology. It is likely that these will be transient and hard to detect as they pass through various stages of the fusion process. This will be markedly simpler if live imaging modalities are developed for both mouse and chicken OFC, but would also benefit from the ability to track these cells in both fixed and live systems by generating fluorescent reporters for pioneer cells. This would also permit their selective isolation from the optic cup enabling single-cell sequencing to define gene expression in those cells that directly mediate fusion.

**Box 1. Summary of outstanding questions for OFC research.**

What are the components of the BM and how are these modulated through the fusion process?

How is the BM remodeled or removed to facilitate fusion?

What are the species-specific requirements for cell death during OFC?

How are pioneer cells specified and what is their fate?

Are pioneer cells essential for fusion in all species?

Do OFM cell protrusions facilitate contact and subsequent fusion?

To what extend do OFM epithelial cells become mesenchymal during fusion and how do these cells subsequently regain epithelial organization?

Thus, we are approaching a point where we may be able to track the gene expression throughout the fusion process. In this context, it is also now theoretically possible to reveal the gene-regulatory networks that govern fusion-specific genes through chromatin capturing studies such as ATACseq or Hi-C, so long as sufficient cell numbers can be isolated. Given the paucity of pioneer cells at the fissure margin in zebrafish, it is likely that only chicken or mouse fissures could currently fulfill this experimental objective. Nevertheless, this will be a powerful approach to reveal conserved genomic regions that regulate OFC genes and complete a comprehensive gene regulatory network for fusion in the eye. Furthermore, this approach could be applied to parallel whole-genome sequencing in human patients to identify non-coding genetic causes of coloboma.

The transcriptomic data that has recently emerged has been matched human coloboma genes to their relevance in wider vertebrate OFC processes. It has also been useful to reveal several novel candidates for the fusion process. Transcriptomic analysis of human OF tissues would be advantageous to augment the available datasets and reinforce the similarities and differences between species, as would repeat transcriptomic analyses of mouse fissures using unbiased RNAseq. Combining all of these data into a meta-analysis of gene-expression in OFC across species would yield insights into both evolutionarily conserved and species-specific differences.

The current studies have also highlighted the limitations to current transcriptomics studies in as far as they have revealed few clear direct clues to how fusion is mediated at the cellular level. This may be due to the limits of detection, e.g., due to there being too few fusion-mediating cells in OF samples with heterogeneous tissues, or it may reflect transcriptional changes that are too subtle or transient to detect. Single-cell analysis, in particular making use of the size advantages of chick eyes or fluorescently marking pioneer cells, may be useful to overcome these problems. However, it may be that the main fusion mediating processes are post-transcriptional and therefore will not be detected by transcriptional analyses. In addition to broad marker analysis using traditional immunolabeling techniques, imaging-coupled proteomic analyses may be a worthwhile avenue to explore. Imaging mass spectrometry (IMS) has been developed for the detection and characterization of spatial abundance patterns for a range of molecule classes in a wide spectrum of tissues. These include small molecule metabolites, lipids, and proteins, and even histone modifications, that can be visualized directly in histological sections of tissues (Ly et al., 2015; Lahiri et al., 2016; Kriegsmann et al., 2019). Advances in technologies such as these open up new ways of understanding the fusion process at the molecular level, outside of the normal constraints of gene and protein analyses. Such techniques could be applied to reveal the compositional nature of the basement membrane and the ECM in the OF. Traditional approaches of ultrastructural analyses are also warranted [e.g., Immuno-gold labeling electron microscopy (EM) analyses] and will be powerful to detect changes to extracellular components during fusion. Serial block face Scanning EM is another technology that would lend itself to OF research as it harnesses the power of ultrastructural analyses by EM with serial-sectioning to yield a 3D reconstruction of the fissure as it fuses. This approach could be used with either model organism or human OFs, if available, and could detail the whole process of fusion at the ultrastructural level. For example, by identifying cellular extensions and their position through basement membranes or between gaps where the BM has been already removed and classifying the position and type/composition of cellular junctions between all of the cell-types at the OF margin.

The requirement for apoptosis or cell death in OFC is still unclear. The available evidence suggests that it is required to maintain an appropriate cell number in the ventral eye, but only in chick eyes are apoptotic foci clearly observed in the fissure margins during normal fusion (Hardy et al., 2019). It would be useful to follow up this observation in the chick to assess its importance through OFC-specific inhibition of apoptosis, e.g., by ectopically expressing the apoptosis inhibitor *Bcl2* or adding caspase-inhibitors in the OF (Fekete et al., 1997; Keoni and Brown, 2015). Similarly, it would be useful to perform comprehensive cell-death assays in human and mouse fissures using histological, TUNEL or immunofluorescence approaches, however, the best data for confirming the requirement will be through human genetics studies -the identification of causative mutations in cell survival factors in coloboma patients.

Through the recent advances described in this review and the promises of powerful tools to augment targeted research, it is now reasonable to predict that the mechanisms for fusion during OFC will be largely elucidated in the near future. Once the main characteristics of this process have been determined at the genetic, molecular and cell-behavioral level, it will be necessary to use this information to determine the other causes of fusion defects—the contribution of environmental factors such as vitamin or nutrient deficiency, maternal illness, or substance abuse (Moosajee and Gregory-Evans, 2006). Having comprehensive datasets and a thorough understanding of fusion, will allow informative studies to test the contributions of these factors, and provide the missing information needed to ultimately lead to the prevention of fusion defects.

## Author Contributions

BC and MM contributed to the content and edited the manuscript. JR conceived and wrote the manuscript and prepared the figures. All authors contributed to the article and approved the submitted version.

## Conflict of Interest

The authors declare that the research was conducted in the absence of any commercial or financial relationships that could be construed as a potential conflict of interest.

## Footnotes

1 https://www.genenames.org/

## References

[B1] Abouzeid H. Boisset G. Favez T. Youssef M. Marzouk I. Shakankiry N. (2011). Mutations in the SPARC-related modular calcium-binding protein 1 gene, SMOC1, cause waardenburg anophthalmia syndrome. *Am. J. Hum. Genet.* 88 92–98. 10.1016/j.ajhg.2010.12.002 21194680 PMC3014360

[B2] ALSomiry A. S. Gregory-Evans C. Y. Gregory-Evans K. (2019). An update on the genetics of ocular coloboma. *Hum. Genet.* 138 865–880. 10.1007/s00439-019-02019-3 31073883

[B3] Barbieri A. M. Lupo G. Bulfone A. Andreazzoli M. Mariani M. Fougerousse F. (1999). A homeobox gene, vax2, controls the patterning of the eye dorsoventral axis. *Proc. Natl. Acad. Sci. U. S. A.* 96 10729–10734. 10.1073/pnas.96.19.10729 10485894 PMC17951

[B4] Bernstein C. S. Anderson M. T. Gohel C. Slater K. Gross J. M. Agarwala S. (2018). The cellular bases of choroid fissure formation and closure. *Dev. Biol.* 440 137–151. 10.1016/j.ydbio.2018.05.010 29803644 PMC7177177

[B5] Božanić D. Tafra R. Saraga-Babić M. (2003). Role of apoptosis and mitosis during human eye development. *Eur. J. Cell Biol.* 82 421–429. 10.1078/0171-9335-00328 14533740

[B6] Brown J. D. Dutta S. Bharti K. Bonner R. F. Munson P. J. Dawid I. B. (2009). Expression profiling during ocular development identifies 2 Nlz genes with a critical role in optic fissure closure. *Proc. Natl. Acad. Sci. U. S. A.* 106 1462–1467. 10.1073/pnas.0812017106 19171890 PMC2631080

[B7] Bush J. O. Jiang R. (2012). Palatogenesis: morphogenetic and molecular mechanisms of secondary palate development. *Development* 139 231–243. 10.1242/dev.079152 22186724 PMC3243091

[B8] Cai Z. Tao C. Li H. Ladher R. Gotoh N. Feng G.-S. (2013). Deficient FGF signaling causes optic nerve dysgenesis and ocular coloboma. *Development* 140 2711–2723. 10.1242/dev.089987 23720040 PMC3678341

[B9] Cao M. Ouyang J. Guo J. Lin S. Chen S. (2018). Metalloproteinase adamts16 is required for proper closure of the optic fissure. *Investig. Ophthalmol. Vis. Sci.* 59 1167–1177. 10.1167/iovs.17-22827 29625437

[B10] Carrara N. Weaver M. Piedade W. P. Vöcking O. Famulski J. K. (2019). Temporal characterization of optic fissure basement membrane composition suggests nidogen may be an initial target of remodeling. *Dev. Biol.* 452 43–54. 10.1016/j.ydbio.2019.04.012 31034836

[B11] Chang L. Blain D. Bertuzzi S. Brooks B. P. (2006). Uveal coloboma: clinical and basic science update. *Curr. Opin. Ophthalmol.* 17 447–470. 10.1097/01.icu.0000243020.82380.f6 16932062

[B12] Chen S. Li H. Gaudenz K. Paulson A. Guo F. Trimble R. (2012). Defective FGF signaling causes coloboma formation and disrupts retinal neurogenesis. *Cell Res.* 23 254–273. 10.1038/cr.2012.150 23147794 PMC3567820

[B13] Chow R. L. Lang R. A. (2001). Early eye development in vertebrates. *Annu. Rev. Cell Dev. Biol.* 17 255–296. 10.1146/annurev.cellbio.17.1.255 11687490

[B14] Davey M. G. Balic A. Rainger J. Sang H. M. McGrew M. J. (2018). Illuminating the chicken model through genetic modification. *Int. J. Dev. Biol.* 62 257–264. 10.1387/ijdb.170323mm 29616734

[B15] Dudas M. Kim J. Li W. Y. Nagy A. Larsson J. Karlsson S. (2006). Epithelial and ectomesenchymal role of the type I TGF-β receptor ALK5 during facial morphogenesis and palatal fusion. *Dev. Biol.* 296 298–314. 10.1016/j.ydbio.2006.05.030 16806156 PMC1557652

[B16] Eckert P. Knickmeyer M. D. Heermann S. (2020). In vivo analysis of optic fissure fusion in zebrafish: pioneer cells, basal lamina, hyaloid vessels, and how fissure fusion is affected by BMP. *Int. J. Mol. Sci.* 21:2760 10.3390/ijms21082760 PMC7215994 32316164

[B17] Fekete D. M. Homburger S. A. Waring M. T. Riedl A. E. Garcia L. F. (1997). Involvement of programmed cell death in morphogenesis of the vertebrate inner ear. *Development* 124 2451–2461. 9199371 10.1242/dev.124.12.2451

[B18] Fitzpatrick D. R. Denhez F. Kondaiah P. Akhurst R. J. (1990). Differential expression of TGF beta isoforms in murine palatogenesis. *Development* 109 585–595. 2401212 10.1242/dev.109.3.585

[B19] Fuhrmann S. (2010). Eye morphogenesis and patterning of the optic vesicle. *Curr. Top Dev. Biol.* 93 61–84. 10.1016/B978-0-12-385044-7.00003-5.Eye 20959163 PMC2958684

[B20] Gato A. Martinez M. L. Tudela C. Alonso I. Moro J. A. Formoso M. A. (2002). TGF-β3-induced chondroitin sulphate proteoglycan mediates palatal shelf adhesion. *Dev. Biol.* 250 393–405. 10.1016/S0012-1606(02)90792-X 12376112

[B21] George A. Cogliati T. Brooks B. P. (2020). Genetics of syndromic ocular coloboma: CHARGE and COACH syndromes. *Exp. Eye Res.* 193:107940. 10.1016/j.exer.2020.107940 32032630 PMC7310839

[B22] Gestri G. Bazin-Lopez N. Scholes C. Wilson S. W. (2018). Cell behaviors during closure of the choroid fissure in the developing eye. *Front. Cell. Neurosci.* 12:42. 10.3389/fncel.2018.00042 29515375 PMC5826230

[B23] Gregory-Evans C. Y. Moosajee M. Hodges M. D. Mackay D. S. Game L. Vargesson N. (2007). SNP genome scanning localizes oto-dental syndrome to chromosome 11q13 and microdeletions at this locus implicate FGF3 in dental and inner-ear disease and FADD in ocular coloboma. *Hum. Mol. Genet.* 16 2482–2493. 10.1093/hmg/ddm204 17656375

[B24] Gregory-Evans C. Y. Williams M. J. Halford S. Gregory-Evans K. (2004). Ocular coloboma: a reassessment in the age of molecular neuroscience. *J. Med. Genet.* 41 881–891. 10.1136/jmg.2004.025494 15591273 PMC1735648

[B25] Haanen C. Vermes I. (1996). Apoptosis: programmed cell death in fetal development. *Eur. J. Obstet. Gynecol. Reprod. Biol.* 64 129–133. 10.1016/0301-2115(95)02261-9 8801138

[B26] Hagedorn E. J. Ziel J. W. Morrissey M. A. Linden L. M. Wang Z. Chi Q. (2013). The netrin receptor DCC focuses invadopodia-driven basement membrane transmigration in vivo. *J. Cell Biol.* 201 903–913. 10.1083/jcb.201301091 23751497 PMC3678161

[B27] Halder G. Dupont S. Piccolo S. (2012). Transduction of mechanical and cytoskeletal cues by YAP and TAZ. *Nat. Rev. Mol. Cell Biol.* 13 591–600. 10.1038/nrm3416 22895435

[B28] Hardy H. Prendergast J. G. Patel A. Dutta S. Trejo-Reveles V. Kroeger H. (2019). Detailed analysis of chick optic fissure closure reveals Netrin-1 as an essential mediator of epithelial fusion. *Elife* 8:e43877. 10.7554/eLife.43877 31162046 PMC6606025

[B29] Hero I. (1989). The optic fissure in the normal and microphthalmic mouse. *Exp. Eye Res.* 49 229–239. 10.1016/0014-4835(89)90093-6 2767170

[B30] Hero I. (1990). Optic fissure closure in the normal cinnamon mouse: an ultrastructural study. *Investig. Ophthalmol. Vis. Sci.* 31 197–216. 2298539

[B31] Ihara S. Hagedorn E. J. Morrissey M. A. Chi Q. Motegi F. Kramer J. M. (2011). Basement membrane sliding and targeted adhesion remodels tissue boundaries during uterine-vulval attachment in Caenorhabditis elegans. *Nat. Cell Biol.* 13 641–651. 10.1038/ncb2233 21572423 PMC3107347

[B32] Iwata J. Parada C. Chai Y. (2011). The mechanism of TGF-β signaling during palate development. *Oral. Dis.* 17 733–744. 10.1111/j.1601-0825.2011.01806.x 21395922 PMC3329177

[B33] Jackson D. Malka S. Harding P. Palma J. Dunbar H. Moosajee M. (2020). Molecular diagnostic challenges for non-retinal developmental eye disorders in the United Kingdom. *Am. J. Med. Genet. Part C Semin. Med. Genet.* 184 578–589. 10.1002/ajmg.c.31837 32830442 PMC8432170

[B34] James A. Lee C. Williams A. M. Angileri K. Lathrop K. L. Gross J. M. (2016). The hyaloid vasculature facilitates basement membrane breakdown during choroid fissure closure in the zebrafish eye. *Dev. Biol.* 419 262–272. 10.1016/j.ydbio.2016.09.008 27634568 PMC5125855

[B35] Kelley L. C. Lohmer L. L. Hagedorn E. J. Sherwood D. R. (2014). Traversing the basement membrane in vivo: a diversity of strategies. *J. Cell Biol.* 204 291–302. 10.1083/jcb.201311112 24493586 PMC3912525

[B36] Keoni C. L. I. Brown T. L. (2015). Inhibition of apoptosis and efficacy of pan caspase inhibitor, Q-VD-OPh, in models of human disease. *J. Cell Death* 8 1–7. 10.4137/JCD.S23844 25922583 PMC4395138

[B37] Knickmeyer M. D. Mateo J. L. Eckert P. Roussa E. Rahhal B. Zuniga A. (2018). TGFb-facilitated optic fissure fusion and the role of bone morphogenetic protein antagonism. *Open Biol.* 8:170134. 10.1098/rsob.170134 29593116 PMC5881030

[B38] Kriegsmann J. Kriegsmann M. Kriegsmann K. Longuespée R. Deininger S. O. Casadonte R. (2019). MALDI imaging for proteomic painting of heterogeneous tissue structures. *Proteomics Clin. Appl.* 13:e1800045. 10.1002/prca.201800045 30471204

[B39] Lahiri S. Sun N. Solis-Mezarino V. Fedisch A. Ninkovic J. Feuchtinger A. (2016). In situ detection of histone variants and modifications in mouse brain using imaging mass spectrometry. *Proteomics* 16 437–447. 10.1002/pmic.201500345 26593131

[B40] Lamouille S. Xu J. Derynck R. (2014). Molecular mechanisms of epithelial–mesenchymal transition. *Nat. Rev. Mol. Cell Biol.* 15 178–196. 10.1038/nrm3758.Molecular 24556840 PMC4240281

[B41] Lee J. Gross J. M. (2007). Laminin β1 and γ1 containing laminins are essential for basement membrane integrity in the zebrafish eye. *Investig. Ophthalmol. Vis. Sci.* 48 2483–2490. 10.1167/iovs.06-1211 17525174

[B42] Lee J. Lee B. K. Gross J. M. (2013). Bcl6a function is required during optic cup formation to prevent p53-dependent apoptosis and colobomata. *Hum. Mol. Genet.* 22 3568–3582. 10.1093/hmg/ddt211 23669349 PMC3736873

[B43] Lupo G. Gestri G. O’Brien M. Denton R. M. Chandraratna R. A. S. Ley S. V. (2011). Retinoic acid receptor signaling regulates choroid fissure closure through independent mechanisms in the ventral optic cup and periocular mesenchyme. *Proc. Natl. Acad. Sci. U. S. A.* 108 8698–8703. 10.1073/pnas.1103802108 21555593 PMC3102374

[B44] Ly A. Schöne C. Becker M. Rattke J. Meding S. Aichler M. (2015). High-resolution MALDI mass spectrometric imaging of lipids in the mammalian retina. *Histochem. Cell Biol.* 143 453–462. 10.1007/s00418-014-1303-1 25534592

[B45] Macdonald R. Scholes J. Strähle U. Brennan C. Holder N. Brand M. (1997). The Pax protein Noi is required for commissural axon pathway formation in the rostral forebrain. *Development* 124 2397–2408. 9199366 10.1242/dev.124.12.2397

[B46] Maeba T. Yonezawa T. Ono M. Tomono Y. Heljasvaara R. Pihlajaniemi T. (2019). Collagen XVIII deposition in the basement membrane zone beneath the newly forming epidermis during wound healing in mice. *Acta Med. Okayama* 73 135–146. 10.18926/AMO/56649 31015748

[B47] Mann I. (1964). *The Development of the Human Eye*, Third Edn London: British Medical Association.

[B48] Mateus R. Holtzer L. Seum C. Hadjivasiliou Z. Dubois M. Jülicher F. (2020). BMP signaling gradient scaling in the zebrafish pectoral fin. *Cell Rep.* 30 4292–4302.e7. 10.1016/j.celrep.2020.03.024 32209485 PMC7109522

[B49] Miesfeld J. B. Gestri G. Clark B. S. Flinn M. A. Poole R. J. Bader J. R. (2015). Yap and Taz regulate retinal pigment epithelial cell fate. *Development* 142 3021–3032. 10.1242/dev.119008 26209646 PMC4582179

[B50] Molè M. A. Galea G. L. Rolo A. Weberling A. Nychyk O. De Castro S. C. (2020). Integrin-Mediated focal anchorage drives epithelial zippering during mouse neural tube closure. *Dev. Cell* 52 321–334.e6. 10.1016/j.devcel.2020.01.012 32049039 PMC7008250

[B51] Moosajee M. Gregory-Evans C. Y. (2006). Advances in the molecular genetics of ocular coloboma. *Expert Rev. Ophthalmol.* 1 209–227. 10.1586/17469899.1.2.209

[B52] Morrison D. FitzPatrick D. Hanson I. Williamson K. van Heyningen V. Fleck B. (2002). National study of microphthalmia, anophthalmia, and coloboma (MAC) in Scotland: investigation of genetic aetiology. *J. Med. Genet.* 39 16–22. 10.1136/jmg.39.1.16 11826019 PMC1734963

[B53] Mui S. H. Kim J. W. Lemke G. Bertuzzi S. (2005). Vax genes ventralize the embryonic eye. *Genes Dev.* 19 1249–1259. 10.1101/gad.1276605 15905411 PMC1132010

[B54] Ng D. Thakker N. Corcoran C. M. Donnai D. Perveen R. Schneider A. (2004). Oculofaciocardiodental and Lenz microphthalmia syndromes result from distinct classes of mutations in BCOR. *Nat. Genet.* 36 411–416. 10.1038/ng1321 15004558

[B55] Nishitani A. M. Ohta S. Yung A. R. del Rio T. Gordon M. I. Abraira V. E. (2017). Distinct functions for netrin 1 in chicken and murine semicircular canal morphogenesis. *Development* 144 3349–3360. 10.1242/dev.144519 28851705 PMC5612249

[B56] Noh H. Lee H. Park E. Park S. (2016). Proper closure of the optic fissure requires ephrin A5-EphB2-JNK signaling. *Development* 143 461–472. 10.1242/dev.129478 26839344

[B57] Okada I. Hamanoue H. Terada K. Tohma T. Megarbane A. Chouery E. (2011). SMOC1 is essential for ocular and limb development in humans and mice. *Am. J. Hum. Genet.* 88 30–41. 10.1016/j.ajhg.2010.11.012 21194678 PMC3014372

[B58] O’Rahilly R. (1966). The early development of the eye in staged human embryos. *Carnegie Instn Wash Publ.* 625 1–42.

[B59] Ozeki H. Ogura Y. Hirabayashi Y. Shimada S. (2000). Apoptosis is associated with formation and persistence of the embryonic fissure. *Curr. Eye Res.* 20 367–372. 10.1076/0271-3683(200005)2051-1FT367 10855031

[B60] Palmer M. A. Nelson C. M. (2017). Epithelial tube fusion as a mechanism for the development of complex lumen-containing organs. *Trends Dev. Biol.* 10 57–69.

[B61] Palmer M. A. Nelson C. M. (2020). Fusion of airways during avian lung development constitutes a novel mechanism for the formation of continuous lumena in multicellular epithelia. *Dev. Dyn.* 249 1318–1333. 10.1002/dvdy.215 32510705

[B62] Patel A. Sowden J. C. (2017). Genes and pathways in optic fissure closure. *Semin. Cell Dev. Biol.* 91 55–65. 10.1016/j.semcdb.2017.10.010 29198497

[B63] Rainger J. van Beusekom E. Ramsay J. K. McKie L. Al-Gazali L. Pallotta R. (2011). Loss of the BMP antagonist, SMOC-1, causes Ophthalmo-acromelic (Waardenburg Anophthalmia) syndrome in humans and mice. *PLoS Genet.* 7:e1002114. 10.1371/journal.pgen.1002114 21750680 PMC3131273

[B64] Rainger J. Williamson K. A. Soares D. C. Truch J. Kurian D. Gillessen-Kaesbach G. (2017). A recurrent de novo mutation in ACTG1 causes isolated ocular coloboma. *Hum. Mutat.* 38 942–946. 10.1002/humu.23246 28493397 PMC5518294

[B65] Ray H. J. Niswander L. (2012). Mechanisms of tissue fusion during development. *Development* 139 1701–1711. 10.1242/dev.068338 22510983 PMC3328173

[B66] Richardson R. Owen N. Young R. M. Tracey-White D. Moosajee M. Toms M. (2019). Transcriptome profiling of zebrafish optic fissure fusion. *Sci. Rep.* 9:1541. 10.1038/s41598-018-38379-5 30733552 PMC6367446

[B67] Rivière J.-B. van Bon B. W. M. Hoischen A. Kholmanskikh S. S. O’Roak B. J. Gilissen C. (2012). De novo mutations in the actin genes ACTB and ACTG1 cause Baraitser-Winter syndrome. *Nat. Genet.* 44 S1–S2. 10.1038/ng.1091 22366783 PMC3677859

[B68] Rolo A. Savery D. Escuin S. de Castro S. C. Armer H. E. J. Munro P. M. G. (2016). Regulation of cell protrusions by small GTPases during fusion of the neural folds. *Elife* 5:e13273. 10.7554/eLife.13273 27114066 PMC4846376

[B69] Sadler T. W. (2010). The embryologic origin of ventral body wall defects. *Semin. Pediatr. Surg.* 19 209–214. 10.1053/j.sempedsurg.2010.03.006 20610194

[B70] Salminen M. Meyer B. I. Bober E. Gruss P. (2000). Netrin 1 is required for semicircular canal formation in the mouse inner ear. *Development* 127 13–22. 10654596 10.1242/dev.127.1.13

[B71] Sanford L. P. Ormsby I. Gittenberger-de Groot A. C. Sariola H. Friedman R. Boivin G. P. (1997). TGFβ2 knockout mice have multiple developmental defects that are non-overlapping with other TGFβ knockout phenotypes. *Development* 124 2659–2670. 9217007 10.1242/dev.124.13.2659 PMC3850286

[B72] Schneiders F. I. Maertens B. Böse K. Li Y. Brunken W. J. Paulsson M. (2007). Binding of netrin-4 to laminin short arms regulates basement membrane assembly. *J. Biol. Chem.* 282 23750–23758. 10.1074/jbc.M703137200 17588941

[B73] Sehgal R. Karcavich R. Carlson S. Belecky-Adams T. L. (2008). Ectopic Pax2 expression in chick ventral optic cup phenocopies loss of Pax2 expression. *Dev. Biol.* 319 23–33. 10.1016/j.ydbio.2008.03.041 18485342 PMC2917900

[B74] Sertié A. L. Sossi V. Camargo A. M. A. Zatz M. Brahe C. Passos-Bueno M. R. (2000). Collagen XVIII, containing an endogenous inhibitor of angiogenesis and tumor growth, plays a critical role in the maintenance of retinal structure and in neural tube closure (Knobloch syndrome). *Hum. Mol. Genet.* 9 2051–2058. 10.1093/hmg/9.13.2051 10942434

[B75] Taylor L. Carlson D. F. Nandi S. Sherman A. Fahrenkrug S. C. McGrew M. J. (2017). Efficient TALEN-mediated gene targeting of chicken primordial germ cells. *Development* 144 928–934. 10.1242/dev.145367 28174243 PMC5374353

[B76] Thomas J. T. Dollins D. E. Andrykovich K. R. Chu T. Stultz B. G. Hursh D. A. (2017). SMOC can act as both an antagonist and an expander of BMP signaling. *Elife* 6:e17935. 10.7554/eLife.17935 28323621 PMC5360445

[B77] Van Der Werff J. F. A. Nievelstein R. A. J. Brands E. Luijsterburg A. J. M. Vermeij-Keers C. (2000). Normal development of the male anterior urethra. *Teratology* 61 172–183. 10.1002/(SICI)1096-9926(200003)61:3<172::AID-TERA4<3.0.CO;2-B 10661906

[B78] Van Nostrand J. L. Brady C. A. Jung H. Fuentes D. R. Kozak M. M. Johnson T. M. (2014). Inappropriate p53 activation during development induces features of CHARGE syndrome. *Nature* 514 228–232. 10.1038/nature13585 25119037 PMC4192026

[B79] Vannahme C. Gösling S. Paulsson M. Maurer P. Hartmann U. (2003). Characterization of SMOC-2, a modular extracellular calcium-binding protein. *Biochem. J.* 373 805–814. 10.1042/BJ20030532 12741954 PMC1223551

[B80] Vannahme C. Smyth N. Miosge N. Gösling S. Frie C. Paulsson M. (2002). Characterization of SMOC-1, a novel modular calcium-binding protein in basement membranes. *J. Biol. Chem.* 277 37977–37986. 10.1074/jbc.M203830200 12130637

[B81] Viringipurampeer I. A. Ferreira T. Demaria S. Yoon J. J. Shan X. Moosajee M. (2012). Pax2 regulates a fadd-dependent molecular switch that drives tissue fusion during eye development. *Hum. Mol. Genet.* 21 2357–2369. 10.1093/hmg/dds056 22357656 PMC3335318

[B82] Vuilleumier R. Springhorn A. Patterson L. Koidl S. Hammerschmidt M. Affolter M. (2010). Control of Dpp morphogen signalling by a secreted feedback regulator. *Nat. Cell Biol.* 12 611–617. 10.1038/ncb2064 20453847

[B83] Weaver M. L. Piedade W. P. Meshram N. N. Famulski J. K. (2020). Hyaloid vasculature and mmp2 activity play a role during optic fissure fusion in zebrafish. *Sci. Rep.* 10:10136. 10.1038/s41598-020-66451-6 32576859 PMC7311462

[B84] Williamson K. A. FitzPatrick D. R. (2014). The genetic architecture of microphthalmia, anophthalmia and coloboma. *Eur. J. Med. Genet.* 57 369–380. 10.1016/j.ejmg.2014.05.002 24859618

[B85] Williamson K. A. Hever A. M. Rainger J. Rogers R. C. Magee A. Fiedler Z. (2006). Mutations in SOX2 cause anophthalmia-esophageal-genital (AEG) syndrome. *Hum. Mol. Genet.* 15 1413–1422. 10.1093/hmg/ddl064 16543359

[B86] Williamson K. A. Rainger J. Floyd J. A. B. Ansari M. Meynert A. Aldridge K. V. (2014). Heterozygous loss-of-function mutations in YAP1 cause both isolated and syndromic optic fissure closure defects. *Am. J. Hum. Genet.* 94 295–302. 10.1016/j.ajhg.2014.01.001 24462371 PMC3928658

[B87] Woodcock M. E. Idoko-Akoh A. McGrew M. J. (2017). Gene editing in birds takes flight. *Mamm. Genome* 28 315–323. 10.1007/s00335-017-9701-z 28612238 PMC5569130

[B88] Yan X. Atorf J. Ramos D. Thiele F. Weber S. Dalke C. (2020). Mutation in BMPR1B leads to optic disc coloboma and ventral retinal gliosis in mice. *Investig. Ophthalmol. Vis. Sci.* 61:44. 10.1167/iovs.61.2.44 32106289 PMC7329948

[B89] Yoon K. H. Fox S. C. Dicipulo R. Lehmann O. J. Waskiewicz A. J. (2020). Ocular coloboma?: Genetic variants reveal a dynamic model of eye development. *Am. J. Med. Genet. Semin. Med. Genet. Part C* 184 590–610. 10.1002/ajmg.c.31831 32852110

[B90] Yung A. R. Nishitani A. M. Goodrich L. V. (2015). Phenotypic analysis of mice completely lacking Netrin-1. *Development* 142 3686–3691. 10.1242/dev.128942 26395479 PMC4647218

[B91] Yurchenco P. D. Schittny J. C. (1990). Molecular architecture of basement membranes. *FASEB J.* 4 1577–1590. 10.1096/fasebj.4.6.2180767 2180767

[B92] Yurchenco P. D. Wadsworth W. G. (2004). Assembly and tissue functions of early embryonic laminins and netrins. *Curr. Opin. Cell Biol.* 16 572–579. 10.1016/j.ceb.2004.07.013 15363809

[B93] Zeisberg M. Hanai J. I. Sugimoto H. Mammoto T. Charytan D. Strutz F. (2003). BMP-7 counteracts TGF-β1-induced epithelial-to-mesenchymal transition and reverses chronic renal injury. *Nat. Med.* 9 964–968. 10.1038/nm888 12808448

